# Low-contrast visual acuity test is associated with central inflammation and predicts disability development in newly diagnosed multiple sclerosis patients

**DOI:** 10.3389/fneur.2024.1326506

**Published:** 2024-02-23

**Authors:** Ettore Dolcetti, Fabio Buttari, Antonio Bruno, Federica Azzolini, Luana Gilio, Veronica Di Caprio, Gianluca Lauritano, Angela Borrelli, Giovanni Galifi, Roberto Furlan, Annamaria Finardi, Alessandra Musella, Livia Guadalupi, Georgia Mandolesi, Valentina Rovella, Diego Centonze, Mario Stampanoni Bassi

**Affiliations:** ^1^Neurology Unit, IRCSS Neuromed, Pozzilli, Italy; ^2^Department of Systems Medicine, Tor Vergata University, Rome, Italy; ^3^Faculty of Psychology, Uninettuno Telematic International University, Rome, Italy; ^4^Clinical Neuroimmunology Unit, Institute of Experimental Neurology (INSpe), Division of Neuroscience, San Raffaele Scientific Institute, Milan, Italy; ^5^Synaptic Immunopathology Lab, IRCCS San Raffaele Roma, Rome, Italy; ^6^Department of Human Sciences and Quality of Life Promotion, University of Rome San Raffaele, Rome, Italy

**Keywords:** LCVA, multiple sclerosis, IL-10, EDSS, MSSS, disability, neuroinflammation

## Abstract

**Introduction:**

The visual system is a prominent site of damage in MS since the earliest phases of the disease. Altered low-contrast visual acuity (LCVA) test has been associated with visual impairment and retinal degeneration, predicting medium- and long-term disability. However, it is unclear whether LCVA may also represent a reliable measure of neuroinflammation and a predictor of disease evolution in the very early stages of MS.

**Methods:**

We explored in a group of 76 consecutive newly diagnosed relapsing–remitting MS (RR-MS) patients without visual impairment or altered visual evoked potentials, the association between LCVA scores at 2.5% and 1.25% and clinical characteristics, including prospective disability evaluated after 1- and 2 years of follow-up. Associations between LCVA and the CSF levels of IL-10 at diagnosis were also analyzed.

**Results:**

A negative correlation was found between LCVA at 2.5% and Expanded Disability Status Scale (EDSS) evaluated at first (Spearman’s Rho = −0.349, *p* = 0.005, *n* = 62) and second year (Spearman’s Rho = −0.418, *p* < 0.001, *n* = 62) of follow-up, and negative correlations were found with Multiple Sclerosis Severity Score (MSSS) at first (Spearman’s Rho = −0.359, *p* = 0.004, *n* = 62) and second year (Spearman’s Rho = −0.472, *p* < 0.001, n = 62). All the data were confirmed by a mixed effect model, considering other clinical variables. A positive correlation was found between the CSF concentrations of IL-10 and LCVA at 2.5% (Spearman’s Rho = 0.272, *p* = 0.020, *n* = 76), and 1.25% (Spearman’s Rho, = 0.276, *p* = 0.018, n = 76), also evidenced in a linear regression.

**Discussion:**

In MS patients at diagnosis, altered LCVA may be associated with CSF inflammation and represent a useful parameter to identify patients with worse disease course.

## 1 Introduction

Multiple Sclerosis (MS) is a chronic autoimmune disease of the CNS characterized by a complex interaction between neuroinflammation and neurodegeneration (1). Increased cerebrospinal fluid (CSF) levels of several proinflammatory cytokines have been associated with MS relapses (2), a more aggressive disease course (3) and higher risk of progression (4). In line with a detrimental role of intrathecal inflammation, previous studies have shown that increased expression of anti-inflammatory molecules may reduce disability accumulation in MS (3, 5). IL-10 represents one of the main anti-inflammatory cytokines (6) and mediates neuroprotective effects in different inflammatory and neurodegenerative conditions (7, 8). Preclinical and clinical studies indicated that this molecule has a crucial role in regulating inflammatory responses in MS (9, 10). Moreover, it has been proposed that IL-10 may contrast inflammatory and degenerative phenomena in MS, exerting a protective effect against disease progression (11, 12).

The visual system represents a prominent site of damage in MS, leading to a wide range of symptoms, including blurred vision and color vision deficits (13). Studies in animal models and in patients with MS have demonstrated that specific proinflammatory molecules directly promote excitotoxic damage (14), and exacerbated CSF inflammation has been associated with worse disease course and higher prospective retinal neurodegeneration (15). Conversely, higher expression of anti-inflammatory molecules may decrease neuronal damage and prevent retinal injury (5).

Low-contrast visual acuity test (LCVA) and specifically Sloan Letter Acuity Chart, which assesses the ability to see objects with low levels of contrast, has been indicated as a measure of visual pathway integrity in MS (13). Accordingly, lower LCVA has been associated with increased axonal damage and demyelination in the optic pathway (16), increased thinning of retinal ganglionic layer (17) and reduced macular volume (18) measured with optical coherence tomography (OCT). While altered LCVA has been consistently associated with retinal degeneration and long-term disability, it is unclear whether it may be associated with CSF inflammation and predict disease course of MS in the very early stages.

In a group of newly diagnosed RR-MS patients, we explored the association between LCVA measured at the time of diagnosis, clinical characteristics, including prospective disability, and the CSF levels of IL-10.

## 2 Materials and methods

### 2.1 MS patients

A group of 76 consecutive RR-MS patients were enrolled in the study. Patients were admitted to the neurological clinic of Neuromed Research Institute in Pozzilli, Italy, between 2016 and 2020 and diagnosed with MS based on McDonald criteria (19). The Ethics Committee of Neuromed Research Institute approved the study (cod. 06-17) according to the Declaration of Helsinki. All patients gave written informed consent to participate in the study. At the time of diagnosis, patients underwent clinical evaluation and brain and spine MRI. Clinical characteristics included age, sex, expanded disability status score (EDSS), the presence of radiological disease activity, and disease duration, measured as the interval between disease onset and performance of specific clinical, neurophysiological, and radiological items used in the study. All patients at the time of enrolment were between 18 and 60 with a disease duration inferior to 5 years, did not experience past episodes of retrobulbar optic neuritis before diagnosis or fully recovered in terms of ophthalmic examination from an episode of optic neuritis, did not suffer from sightedness pathology (severe myopia or strabismus) and did not present latent optic pathway damage detected at visual evoked potentials (PEV) at diagnosis. EDSS visual FS score at diagnosis was 0 for all the subjects. Patients with uncomplete recovery from past episodes of optic neuritis were excluded. In 62 patients we evaluated prospective EDSS and Multiple Sclerosis Status Scale (MSSS) after 1 and 2 years of follow-up. Patients had not received corticosteroids or immunoactive therapies before enrolment. Disease modifying therapies (DMTs) were started after diagnosis as follows: platform therapies (interferon beta-1a 30 mcg = 1 patient; interferon beta-1a 22 mcg = 4 patients; interferon beta-1a 44 mcg = 1 patient; peginterferon beta-1a = 3 patients; teriflunomide = 3 patients; glatiramer acetate = 11 patients; dimethyl fumarate = 29 patients; total 52), high-efficacy therapies (ocrelizumab = 2 patients; cladribine = 5 patients; fingolimod = 10 patients; natalizumab = 6 patients; rituximab = 1 patient; total 24). DMTs were selected according to practical guidelines.

### 2.2 LCVA analysis

In all the patients, clinical assessments were performed at the time of diagnosis, carrying on all patients low-contrast visual acuity measurements at 2.5% (seen letters) and 1.25% (seen letters), averaged between the two eyes, at low-contrast Sloan letter chart, Precision Vision, LaSalle, IL (20).

### 2.3 CSF collection and analysis

In 73 patients CSF concentrations of IL-10 were analyzed. CSF was collected at the time of diagnosis, during hospitalization, by lumbar puncture (LP). CSF samples were stored at −80°C and later analyzed using a Bio-Plex multiplex cytokine assay (Bio-Rad Laboratories, Hercules, CA, United States). CSF IL-10 levels were determined according to a standard curve generated for the specific target and expressed as picograms/milliliter (pg/mL). Samples were analyzed in triplicate.

### 2.4 MRI

All the patients underwent a 1.5 T MRI scan, which included the following sequences: dual-echo proton density, fluid-attenuated inversion recovery (FLAIR), T1-weighted spin-echo (SE), T2-weighted fast SE, and contrast-enhanced T1-weighted SE before and after intravenous gadolinium (Gd) infusion (0.2 mL/kg). Radiological disease activity at the time of diagnosis was defined as the presence of Gd-enhancing (Gd+) lesions at the time of hospitalization.

### 2.5 Statistical analysis

Spearman’s nonparametric correlation and partial correlation were used to test possible associations between variables that were not normally distributed. A *p*-value ≤ 0.05 was considered statistically significant. All the comparisons were performed using IBM SPSS Statistics for Windows/Mac (IBM Corp., Armonk, NY, United States). Linear regression and mixed model effect analyses were undertaken to explore the association between EDSS at first and second year as dependent variable and multiple potential confounding factors (age, gender, disease duration, disease-modifying treatments, EDSS at baseline, clinical relapses and radiological activity at follow-up, and LCVA measurements).

## 3 Results

### 3.1 LCVA evaluated at the time of diagnosis is associated with prospective disability in RR-MS

The demographic and clinical characteristics of patients are shown in Tables 1, 2.

**Table 1:** Clinical characteristics of MS patients at baseline.

RR-MS (76)	
Age, years (mean, SD)	34.6 ± 10.3
Sex, F/M (*N*, %)	59/17 (77.6)
Disease duration, months (median, IQR)	3.6 (1.2–19.3)
Radiological activity (1 = yes, 0 = no) (N/tot (%))	37/74 (50)^*^
EDSS (median, IQR)	1.5 (1–2)
MSSS (median, IQR)	3.9 (2.9–6.5)
LCVA at 2.5% (median, IQR)	43 (38–45)
LCVA at 1.25% (median, IQR)	37 (32–42)
CSF IL-10 (pg/mL) (mean, SD)	2.3 ± 1.8
DMT (0 = platform, 1 = high efficacy) (*N*, %)	52/24 (68.4)

SD, standard deviation; N, number; IQR, interquartile range; EDSS, Expanded Disability Status Scale; MSSS, Multiple Sclerosis Severity Scale; LCVA, low-contrast visual acuity; CSF, cerebrospinal fluid; DMT, disease-modifying therapies; platform therapies: interferon beta-1a 30 mcg = 1 patient; interferon beta-1a 22 mcg = 4 patients; interferon beta-1a 44 mcg = 1 patient; peginterferon beta-1a = 3 patients; teriflunomide = 3 patients; glatiramer acetate = 11 patients; dimethyl fumarate = 29 patients; total 52; high-efficacy therapies (ocrelizumab = 2 patients; cladribine = 5 patients; fingolimod = 10 patients; natalizumab = 6 patients; rituximab = 1 patient; total 24). ^*^Missing data for 2 patients.

**Figure 1:**
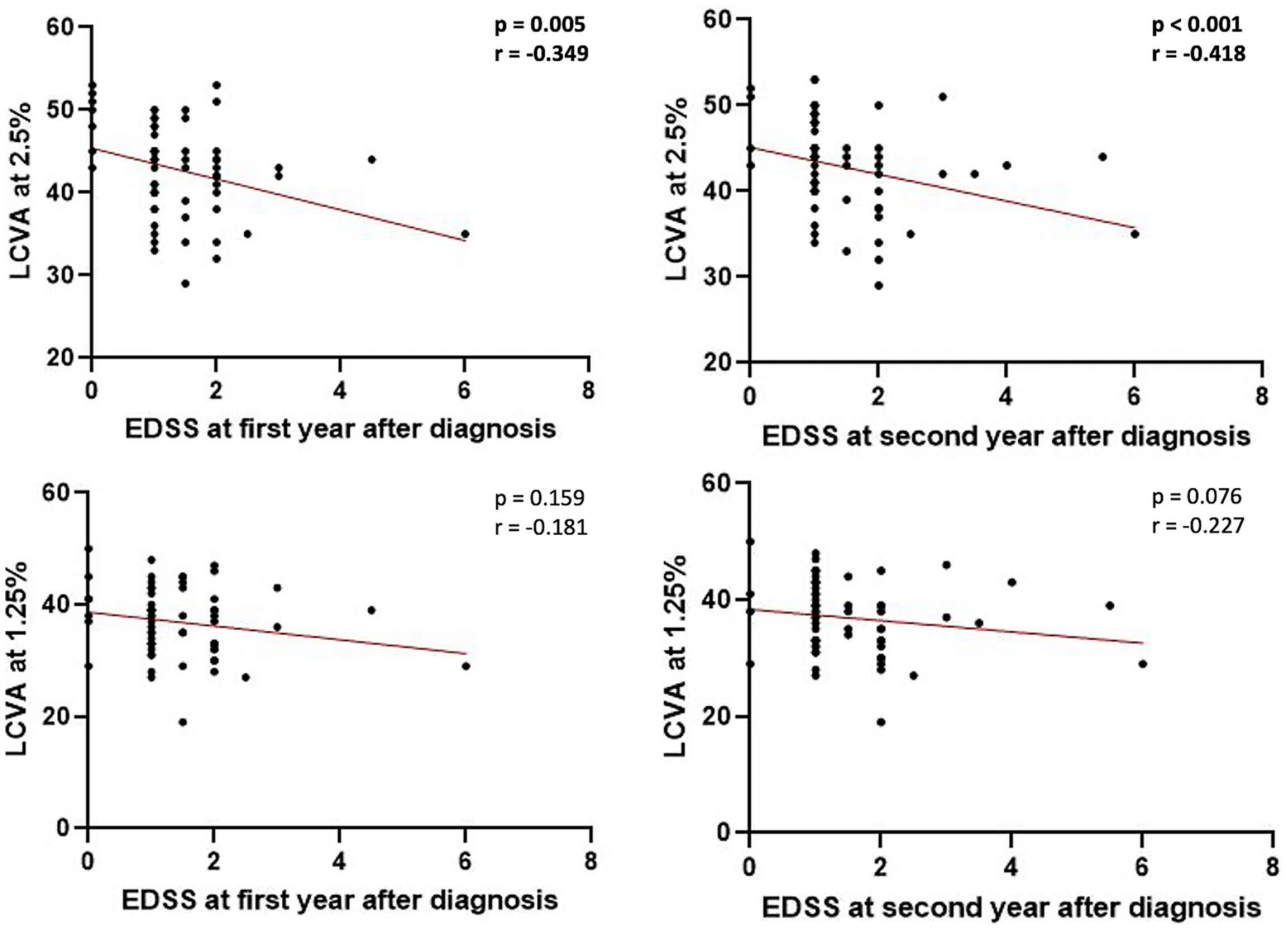
Correlations between EDSS at first and second year after diagnosis and LCVA at 2.5% and 1.25%. LCVA, low-contrast visual acuity; EDSS, Expanded Disability Status Scale; bold denotes statistical significance.

**Figure 2:**
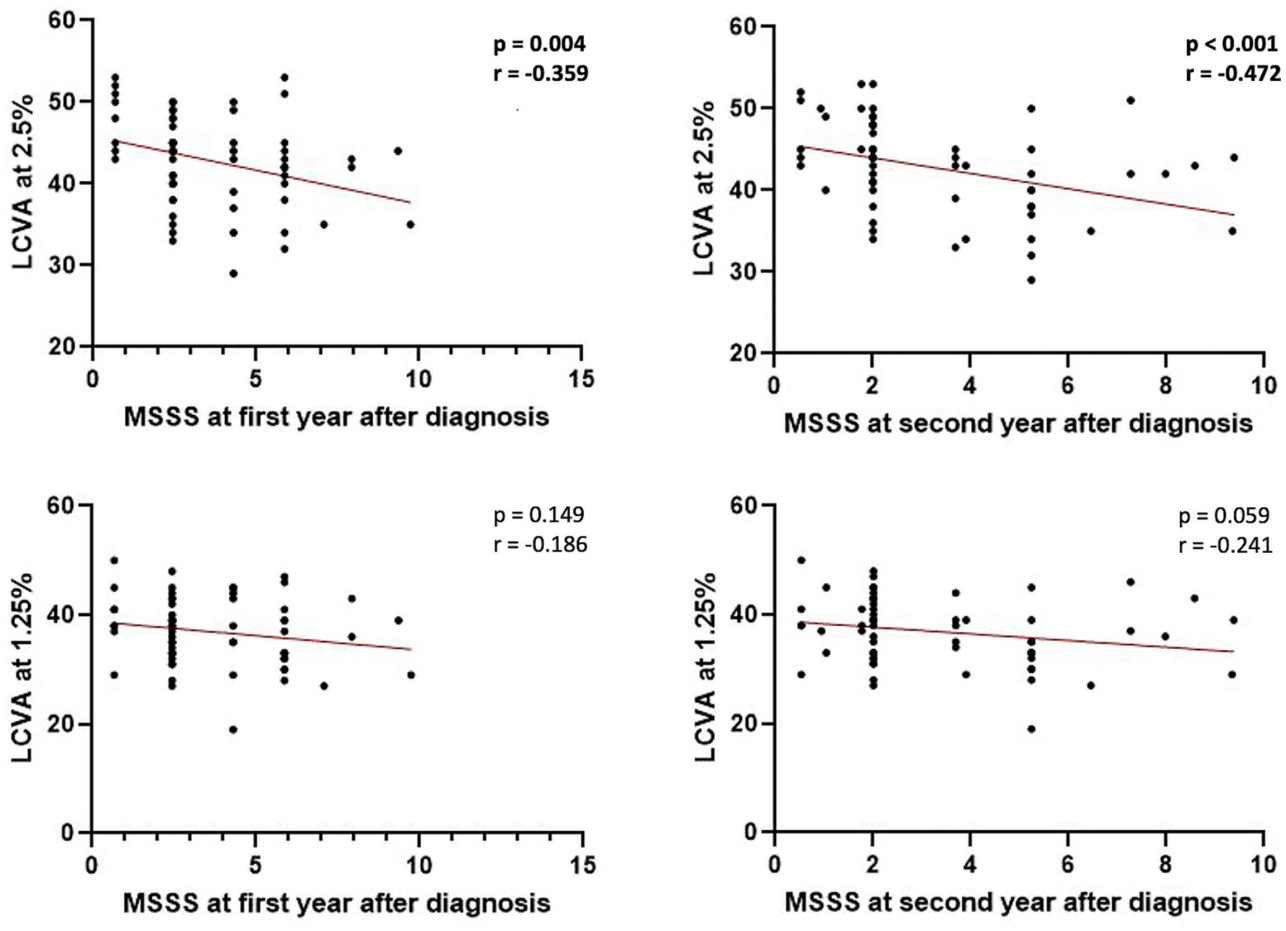
Correlations between MSSS at first and second year after diagnosis and LCVA at 2.5% and 1.25%. LCVA, low-contrast visual acuity; MSSS, Multiple Sclerosis Severity Scale; bold denotes statistical significance.

No significant associations were found between LCVA at 2.5% and 1.25% charts and clinical characteristics at the time of diagnosis. A negative correlation emerged between LCVA values at 2.5% chart and EDSS evaluated after 1 year of follow-up (Spearman’s Rho = −0.349, *p* = 0.005, *n* = 62) and after 2 years of follow-up (Spearman’s Rho = −0.418, *p* < 0.001, *n* = 62; Figure 1). Moreover, we found a negative correlation between LCVA at 2.5% and MSSS (MSSS first year: Spearman’s Rho = −0.359, *p* = 0.004, n = 62; MSSS second year: Spearman’s Rho = −0.472, *p* < 0.001, *n* = 62; Figure 2). In our cohort, EDSS at baseline correlates with EDSS at first (Spearman’s linear correlation, r = 0.497, *p* < 0.001) and second year (Spearman’s linear correlation, r = 0.402, *p* = 0.001), as already known in previous studies (21, 22). Then we performed a mixed effect model to evaluate association between LCVA at 2.5% and disability measures at follow-up, considering also the effect of other clinical variables (age, sex, disease duration, platform or high-efficacy DMT, EDSS at baseline, clinical relapses, presence of new T2 lesions at MRI); EDSS at first year: LCVA at 2.5% (coefficient − 0.048, t = −2.372, 95% CI −0.089 to −0.007, *p* = 0.021); EDSS at second year: LCVA at 2.5% (coefficient − 0.060, t = −2.446, 95% CI −0.109 to −0.010, *p* = 0.019); MSSS at first year: LCVA at 2.5% (coefficient −0.123, t = −2.805, 95% CI −0.211 to −0.035, *p* = 0.007); MSSS at second year: LCVA at 2.5% (coefficient − 0.130, t = −2.697, 95% CI −0.226 to −0.033, *p* = 0.009).

**Table 2:** Clinical characteristics of MS patients at follow-up.

RR-MS (62)	
EDSS at first year (median, IQR)	1 (1–2)
EDSS at second year (median, IQR)	1 (1–2)
MSSS at first year (median, IQR)	2.4 (2.4–5.9)
MSSS at second year (median, IQR)	2 (2–5.2)
Clinical activity in the first year (1 = yes, 0 = no), (N/tot (%))	7/62 (11.3)
Radiological activity in the first year (1 = yes, 0 = no), (N/tot (%))	29/62 (46.8)
Clinical activity in the second year (1 = yes, 0 = no) (N/tot (%))	3/62 (4.8)
Radiological activity in the second year (1 = yes, 0 = no), (N/tot (%))	11/62 (17.7)

IQR, interquartile range; EDSS, Expanded Disability Status Scale; MSSS, Multiple Sclerosis Severity Scale.

No significant correlations emerged between LCVA at 1.25% chart and EDSS at first (*p* = 0.159) and second year (*p* = 0.076; Figure 1), and likewise between LCVA at 1.25% and MSSS at first (*p* = 0.149) and second year (*p* = 0.059) after diagnosis (Figure 2).

### 3.2 LCVA represents a parameter of low central inflammation in MS patients

**Figure 3:**
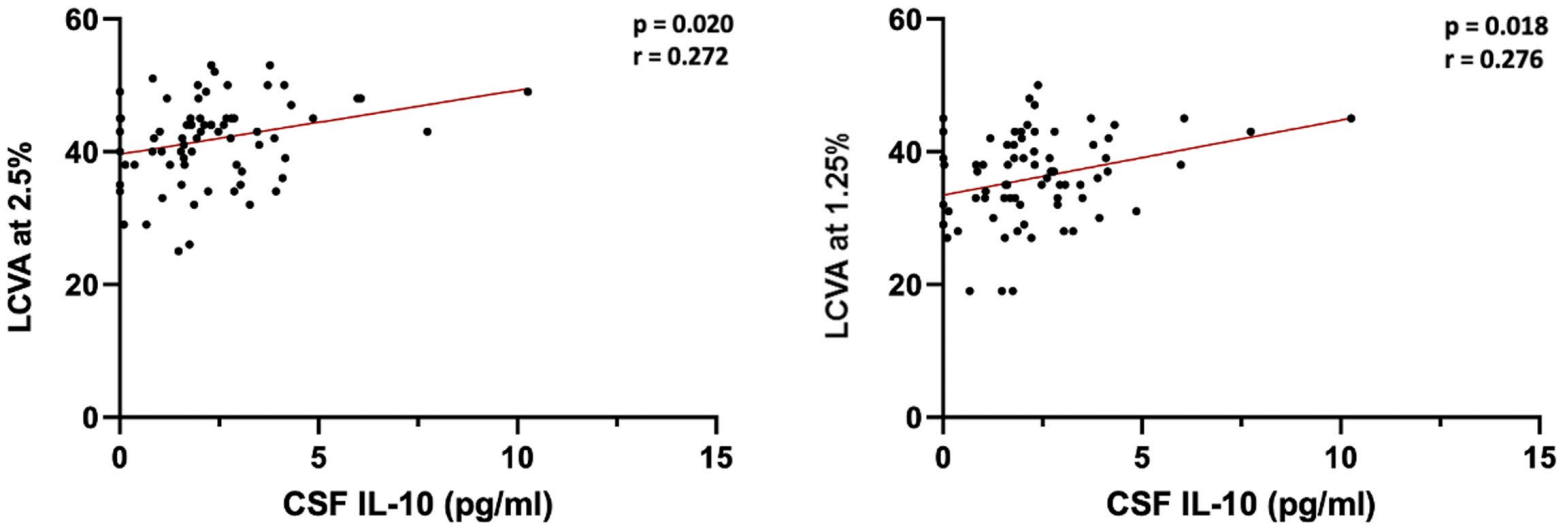
Correlations between CSF IL-10 and LCVA at 2.5% and 1.25%. LCVA, low-contrast visual acuity; CSF, cerebrospinal fluid; bold denotes statistical significance.

We evaluated whether LCVA could be associated with intrathecal levels of IL-10 at the time of diagnosis in MS patients. In our population we did not find associations between CSF IL-10 levels and disability measured by EDSS at baseline (*p* = 0.493) and over first (*p* = 0.257) and second year (*p* = 0.404) after diagnosis. A positive correlation between CSF IL-10 and LCVA at 2.5% (Spearman’s Rho = 0.272, *p* = 0.020, *n* = 76) and at 1.25% chart (Spearman’s Rho, coefficient of correlation 0.276, *p* = 0.018, *n* = 76) was found (Figure 3). We confirmed data performing a linear regression, adjusted for age, sex and disease duration [CSF IL-10 levels: LCVA at 2.5% (Beta = 0.347, 95% CI 0.289–2.107, *p* = 0.011; CSF IL-10 levels: LCVA at 1.25% (Beta = 0.346, 95% CI 0.405–2.187, *p* = 0.005))].

## 4 Discussion

Inflammatory neurodegeneration may play a crucial role in MS progression (23). Accordingly, increased neuronal atrophy and higher CSF inflammation at the time of MS diagnosis have been associated with higher prospective disability and increased risk of progression (24–26).

Reduced visual acuity to low-contrast stimuli represents a sensible marker of retinal involvement in MS (27), and a useful tool to identify disease progression (28). Several studies have shown that LCVA can detect subclinical visual impairment in patients with MS (29), and lower LCVA performance has been associated with decreased ganglion cell thickness at OCT, abnormal responses to visual evoked potentials, and worse electrophysiological signature patterns at electroretinogram (30, 31).

In our study, in a group of RR-MS patients, we found an association between LCVA measured at the time of diagnosis and prospective disability accumulation. Better performance at LCVA 2.5% chart predicted lower EDSS and MSSS scores assessed after 1 and 2 years of follow-up. These data are in line with previous studies evidencing that reduced LCVA values correlated with higher EDSS and increased risk of visual impairment in the course of the disease (32). Furthermore, worsening of LCVA has been associated with increased disability in medium term (33), and its alteration can anticipate disease relapses by several months (33). In our cohort, although a negative trend was observed between LCVA at 1.25% and EDSS and MSSS, no significant correlations were found. Previous studies have shown that LCVA 2.5 and 1.25% chart similarly predict disability worsening, although some discrepancies exist between the two tests (34), and particularly the 1.25% chart may have a higher floor effect.

Despite considerable evidence indicates the role of LCVA as a prognostic tool in MS, it has not yet been clarified whether this parameter may also represent an indirect index of neuroinflammation (33).

We explored, at the time of MS diagnosis, the correlation between LCVA and the CSF concentration of the anti-inflammatory cytokine IL-10. A positive correlation emerged between IL-10 and LCVA at 2.5% and 1.25% charts, suggesting that higher CSF expression of this molecule in the early MS phases could be associated with better LCVA performance.

IL-10 is a main anti-inflammatory cytokine which regulates adaptive immune response by modulating Th1 cell and macrophages activation and reducing the expression of various proinflammatory mediators (35). A role of IL-10 in the pathogenesis of MS has been consistently evidenced in experimental studies based on animal models of MS (i.e., experimental autoimmune encephalomyelitis, EAE), and in humans. Increased clinical deterioration and neurodegeneration have been reported in EAE mice lacking IL-10 (36–39). Moreover, reduced CSF concentrations of IL-10 were detected in MS patients comparing with control subjects (40). The expression of this molecule is decreased during acute relapses and increased in stable disease phases (10, 41), and higher levels of IL-10 have been associated with a reduced disability accumulation (42).

Our data are in line with a protective role of IL-10 in the early phases of MS. Evidence from preclinical studies suggest that anti-inflammatory molecules may have a protective role on inflammatory retinal neurodegeneration. It has been evidenced that higher CSF levels of the anti-inflammatory molecule IL-13 may contrast synaptic alterations in MS and reduce retinal atrophy assessed with OCT (5). Also, IL-10 has been associated with neuroprotective effects in different inflammatory and degenerative conditions (7) directly modulating neuronal activity (7). Accordingly, IL-10 receptors are expressed by astrocytes, oligodendrocytes, and neurons (43, 44) and experimental studies evidenced that this molecule may contrast neuronal hyperexcitability regulating synaptic plasticity (45), and preventing excitotoxic damage (46, 47).

Our findings confirm that LCVA may be a sensitive marker of prospective disability accumulation even in the earliest stages of MS. Furthermore, our results suggest that LCVA alteration could be an expression of increased central inflammation, making it a useful tool to monitor inflammatory processes in MS and to identify patients at risk of progression.

Associations between LCVA and EDSS has been previously investigated in heterogeneous MS patient populations (18, 33), here we included patients at the time of diagnosis, with low disease duration, young median age, and free from subclinical alteration of visual evoked potentials (VEP). The low sample size and the lack of imaging measures of neurodegeneration represent important limitations of the present study, as well as lack of data on LCVA follow-up. Single-eye LCVA analysis cannot be performed since the measurements are normalized for binocular vision. Moreover, as prospective LCVA evaluation over time was not available, further studies are needed to explore the association between IL-10 CSF concentrations at the time of diagnosis and LCVA performance during the follow-up.

In conclusion, altered LCVA represents a marker of prospective disability accumulation in the early phases of MS and may be associated with increased CSF inflammation. Identification of neuroinflammatory phenomena, even subclinical, may be important to recognize patients at increased risk of progression and therefore candidates for high-efficacy therapies.

## Data availability statement

The raw data supporting the conclusions of this article will be made available by the authors, without undue reservation.

## Ethics statement

The studies involving humans were approved by Neuromed Research Institute Ethics Committee. The studies were conducted in accordance with the local legislation and institutional requirements. The participants provided their written informed consent to participate in this study.

## Author contributions

ED: Conceptualization, Formal analysis, Writing – original draft, Writing – review & editing. FB: Conceptualization, Funding acquisition, Writing – review & editing. ABr: Data curation, Writing – original draft. FA: Data curation, Writing – original draft. LGi: Data curation, Writing – original draft. VC: Data curation, Writing – original draft. GL: Data curation, Writing – original draft. ABo: Writing – original draft. GG: Data curation, Writing – original draft. RF: Data curation, Writing – original draft. AF: Data curation, Writing – original draft. AM: Data curation, Writing – original draft. LGu: Data curation, Writing – original draft. GM: Data curation, Funding acquisition, Writing – original draft. VR: Data curation, Data curation. DC: Funding acquisition, Writing – review & editing, Conceptualization. MS: Formal analysis, Funding acquisition, Writing – original draft, Writing – review & editing.
